# Feasibility of a Home-Based Action Observation Training for Children With Unilateral Cerebral Palsy: An Explorative Study

**DOI:** 10.3389/fneur.2020.00016

**Published:** 2020-02-28

**Authors:** Elena Beani, Valentina Menici, Adriano Ferrari, Giovanni Cioni, Giuseppina Sgandurra

**Affiliations:** ^1^Department of Developmental Neuroscience, IRCCS Fondazione Stella Maris, Pisa, Italy; ^2^Children Rehabilitation Unit, Azienda USL - IRCCS Reggio Emilia, Reggio Emilia, Italy; ^3^Department of Neuroscience, University of Modena and Reggio Emilia, Modena, Italy; ^4^Department of Clinical and Experimental Medicine, University of Pisa, Pisa, Italy

**Keywords:** feasibility, home-based training, action-observation training, upper limb, unilateral cerebral palsy, children

## Abstract

Unilateral Cerebral Palsy (UCP), the most frequent form of Cerebral Palsy, usually affects more the upper limb (UL) than the lower limb. Rehabilitation programs are addressed to improve manual abilities and UL use. In recent years, Information and Communication Technology (ICT) has been introduced in rehabilitation to increase treatment opportunities for patients, and also in home-based intervention. Moreover, the discovery of the Mirror Neuron System allowed to insert a new paradigm of treatment that is the Action Observation Training (AOT). The aim of the present study was to investigate the feasibility of a new rehabilitative home-based approach, called Tele-UPCAT (Tele-monitored UPper Limb Children Action Observation Training), based on the principles of AOT, in a group of Italian children and adolescents with UCP. This investigation was to provide information about the possibility of introducing ICT in telerehabilitation field. Twenty-nine children aged 11.73 ± 3.65 years (range 6.00–18.75) with a diagnosis of UCP participated in the study. They carried out 15 days of training based on the AOT paradigm with Tele-UPCAT system while wearing Actigraphs on both wrists. The feasibility of both training and study design and procedures was assessed through nine criteria taken from existent literature and from a questionnaire designed and realized *ad hoc* for the purpose, based on standard items of usability and acceptability. All feasibility criteria were met: 80% of training sessions were completed in the planned time and no significant technical issues were found. From the questionnaire, total scores were all above 82.15%, while the four sections obtained the following scores: (i) customization of exercises 80.00%; (ii) acceptability at home, 77.50%; (iii) required effort 80.00%; and (iv) suitability of manual and software 95.00%. No differences were found for age and sex. Tele-UPCAT demonstrated to be feasible as a home-based AOT for children and adolescents with UCP. Trial registration NCT03094455.

## Introduction

Unilateral Cerebral Palsy (UCP) is the most frequent form of Cerebral Palsy, representing 30–40% of all affected children (1, 2). Due to the fact that in the majority of cases the upper limb (UL) is more affected than the lower limb (LL), rehabilitation programs are mainly addressed to improve manual abilities and UL use and integration. Together with the traditional methods of rehabilitative intervention, the discovery of the Mirror Neuron System allowed to insert a new paradigm of treatment, that is, the Action Observation Training (AOT) (3). The AOT is based on neurophysiological knowledge that the observation of a goal-directed action activates the same neural substrate, the Mirror Neuron System, as the physical execution of the same action (4, 5). AOT evidence is rising in literature, mainly in adult population, but in some works, it has been used also in pediatric samples (6–10).

Beside face-to-face trials, the recent introduction of Information and Communication Technologies (ICTs) has increased treatment opportunities for patients directly at home. Home-based therapies, defined as “therapeutic activities that the child performs with parental assistance in the home environment with the goal of achieving desired health outcomes” (11), represent a way to facilitate children and adolescent access to rehabilitation and to enhance motivation.

Innovative rehabilitation trainings based on ICT devices have been introduced to increase opportunities and to add objective data in rehabilitation. The use of ICT for rehabilitation purposes at home is referred to as telerehabilitation (12, 13). Telerehabilitation allows care continuity and limits time and economic demands for families and institutes. Moreover, it enables precise monitoring of patients' performance through online tracking (14, 15).

From the union between the innovative paradigm of treatment that is the AOT and the innovative approach represented by the home-based therapy, the Tele-UPCAT (Tele-monitored UPper Limb Children Action Observation Training), built by the BioRobotics Institute of Scuola Superiore Sant'Anna in collaboration with IRCCS Fondazione Stella Maris, has been recently introduced in the panorama of rehabilitative proposals for children with UCP (16).

Tele-UPCAT is a platform to practice AOT at home, designed to be user-friendly both for children and adolescents, at home in a playful setting with integrated smart features. In fact, two age-related interfaces have been developed, following the interest and motivational factors of the two age ranges; moreover, the management of the entire system is very simple for families, as the software has an automatic process of execution and continuation of the program just needs to push a button; finally, the integrated camera has a reminder for being turned on and off.

Given the economic and energy advantages that home therapy brings, the issues of usability and acceptability of a technology-based therapy into the home environment need to be investigated. It is crucial to investigate the end-user opinions during the use at home of a new tool. A new home rehabilitation system could be effective, but its use could be not feasible from the user's point of view; this highlights the importance of evaluating the feasibility aspects before evaluating the effectiveness.

Based on these aspects, the aim of the present study is to investigate the feasibility, acceptability, and usability of the Tele-UPCAT in a group of Italian children and adolescents with UCP. This investigation will provide information about the possibility of introducing ICT in the telerehabilitation field.

## Materials and Methods

This study was a part of a wider study, aimed to investigate if a new Information and Communication Technology platform, called Tele-UPCAT, could be able to deliver the AOT in a home setting and test its feasibility and efficacy in children and adolescents with UCP.

The study has been approved by Tuscany Pediatric Ethics Committee (169/2016), and it was registered (NCT03094455) on Clinical Trials.gov.

According to CONSORT guidelines (17), the sample size estimate has been based on projected treatment effect on the primary outcome measure, the Assisting Hand Assessment (AHA). Taking into account the study design and the stratification, a minimum sample size of 10 per group was required in order to detect a 1.40 effect size [the value based on our preliminary data; (8, 18)] at a significant level of 0.05% and 80% power. Considering 20% of possible dropouts, a minimum of 12 participants per group were recruited, with a total sample of 24 participants.

Protocol and system description, together with the psychometric properties of the selected outcome measures, have been already extensively reported elsewhere (16). Briefly, the study has been designed as a randomized, allocation concealed (waitlist control) and evaluator-blinded clinical trial with two investigative arms, which were AOT vs. standard care (SC). Both groups are assessed with clinical tools (Assisting Hand Assessment, Melbourne Assessment 2, Box and Block Test, ABILHAND-Kids, Participation and Environment Measure—Children and Youth and Cerebral Palsy Quality of Life Questionnaire) and technological outcome measures (a purpose-designed sensorized toy and Actigraphs). The experimental group, after the first assessment (T0), proceeded with a home-based 3-week AOT with the Tele-UPCAT system, then the assessment is repeated immediately after this period (T1) and after 8 (T2) and 24 (T3) weeks after the end of the training. The control group starts with a 3-week period of SC, then follows the same pathway as the experimental group, which meant the AOT and the follow-up assessments. Parents of the enrolled participants were asked to provide written informed consent in order to allow data collection and analysis for study purposes. All parents provided informed consent. For subjects of ages above 12 years, an additional consensus has been asked to the child, with a dedicated form.

### Participants

Participants were recruited among children and adolescents with congenital UCP referring to IRCCS Fondazione Stella Maris (Pisa) and Unit of Children Rehabilitation of S. Maria Nuova Hospital (Reggio Emilia).

The main inclusion criteria were as follows: age between 5 and 20 years, confirmed diagnosis of spastic motor type of UCP, minimum ability of manual function defined as the ability to passively hold an object placed in the hand or hold and stabilize an object with a hand while the other manipulates it (i.e., House Functional Classification System, HFCS ≥ 2), a normal cognitive level (i.e., IQ ≥ 70), and no disabling behavioral disorders. The main exclusion criteria were as follows: previous orthopedic surgery or botulinum toxin A (BoNT-A) injection in the UL within 6 months prior to the enrolment of this study.

Patients' recruitment was conducted by a research team member who assisted, when necessary and requested, families during the AOT and proposed to fill in a questionnaire specifically designed for the purpose (described below) of assessing the feasibility at the end of the training. In case of assent, parents and/or children were requested to complete the questionnaire in the printed or electronic version.

Recruitment for this preliminary study on feasibility started after the approval of the research project by the Ethics Committee.

### Study Design and Procedure

This study represents a part of the Tele-UPCAT trial, an exploratory randomized, allocation concealed (waitlist controlled) and evaluator-blinded clinical trial with two investigative arms using an AOT intensive rehabilitation program of home-based AOT compared with SC in children and adolescents with UCP, whose recruitment started on March 2017 and was completed on November 2018. The total minimum sample size was planned for 24 children.

As shown in the study design (16), participants were assessed at baseline and randomly allocated to the immediate AOT group or waitlist SC.

The primary outcome measure was the AHA. The Melbourne Assessment 2, Box and Block Test, ABILHAND-Kids, Participation and Environment Measure—Children and Youth, and Cerebral Palsy Quality of Life Questionnaire were included as secondary measures. In addition, quantitative measures from the available ICT were added, i.e., the assessment with a sensorized object and two wearable sensors on both wrists (Actigraphs GXT3+), used both during the assessments and in the intervention period (AOT and SC). The assessment points were the week before (T0) and after (T1) the period of AOT/SC. Further assessments were then carried out in the week after the AOT period for the waitlist group (T1 plus) and at 8 weeks (T2) and 24 weeks (T3) after the AOT.

Concerning the intervention, the experimental group performed the AOT at home for 3 weeks using a customized Tele-UPCAT system. Participants were asked to complete 15 sessions of the AOT at home for 45 to 60 min per working day for 3 weeks (5 days per week). Through the Tele-UPCAT system, they watched 3-min first-person video sequences of unimanual or bimanual goal-directed actions, and then they had to execute the same action for 3 min. Three different actions were proposed twice each day. The type and the order of the games were identical for all participants in terms of actions (unilateral or bilateral and increasing complexity) but variable in terms of objects (the same goal but different shape of toys) in order to be suitable for different hand abilities.

The Tele-UPCAT platform has been delivered to the participant's home by the engineers in charge of system installation together with a research team member, who assisted families for the beginning of the training. Weekly telephone-based contact with the participants and their parents was then conducted by the research team member, with the aim of assisting them for the training, sustaining training compliance and motivation, and recording the reasons of any eventual issue.

The control group received usual care for 3 weeks, which might include UL training, and then they were asked to receive the AOT at home by means of the Tele-UPCAT system after 3 weeks. In each case, participants were asked to wear an Actigraph at both wrists to measure the UL mean activity and to fill in a daily diary with notes related to the time of main activities of the day and the time with and without Actigraphs. In this way, some quantitative data were added not only during the assessments but also during the 3 week period of training and SC.

Data on training performance were collected on a remote database available at IRCCS Fondazione Stella Maris. The number of days for completing the program (15 days of training) and effective time with/without wearing Actigraphs were recorded for each patient. An *ad hoc* questionnaire has been created and administered to all participants at the post-training assessment (T1 for the AOT group and T1 plus for the control group) by an operator who structured it as an interview to the child/adolescents with the presence of parents.

### Intervention

**Figure 1:**
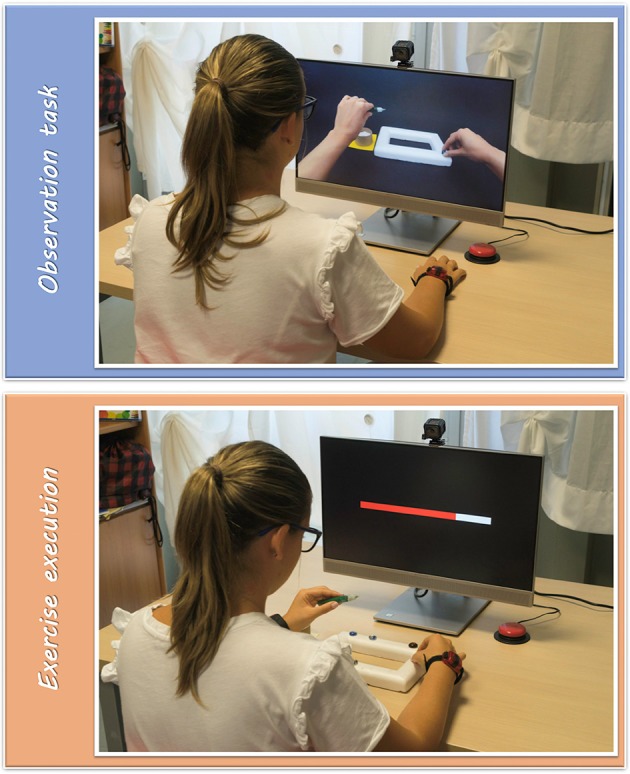
The Tele-UPCAT system.

Tele-UPCAT system (Figure 1) is composed of two different modules: *the observation module (OM)*, which is mainly dedicated to the presentation of videos for the “observational task,” and the *motor performance module (MPM)*, for the “exercise execution,” which consists of objects and toys for executing the action observed in videos and two Actigraphs (wGT3X-BT) for recording UL activities.

All the actions were chosen based on some daily activities, goal-directed to meaningful actions of children and adolescents that evocate hand and arm movements to be stimulated because of their impairment due to the UCP. In the first half of the sessions, movements require the use of the affected side and, in the second half, the bimanual cooperation. The selected objects are mainly toys (e.g., some glitter glue with some marbles to be attached to a plastic frame) or tools that remind one of some daily objects (such as a bottle and a glass). Details of actions of the training are presented in the study protocol description (16).

Each daily session is composed of three goal-directed actions with increasing difficulty within days of the training. As described above, the training is planned in 15 sessions to be performed once a day in 3 weeks, 5 days a week.

Each activity is presented with an age-appropriate software package for motivating participants and explaining the training rules: the cartoon with the adventures of an explorer called Ubi and its missions in the galaxy for children aged <12 years and a PowerPoint presentation with simple slides and voice guide for adolescents (>12 years).

### Outcome Measures

The feasibility of the AOT with Tele-UPCAT was investigated by three measures: the acceptability and usability from the questionnaire, the criteria for the feasibility taken from the literature, and the acceptability in terms of wearing time of Actigraphs.

First, the study of the acceptability and usability was assessed through the *ad hoc* purpose-designed questionnaire (widely described below). According to the study design (16), the questionnaire was administrated during the post-training assessment (T1 or T1 plus for the immediate AOT and the waitlist group, respectively).

Moreover, feasibility outcome measures were taken from the literature (19–21), and they are criteria based on relevant recommendations for conducting research on feasibility. In details, there are nine feasibility measures: four relative to training intervention (accessibility, training motivation, technical smoothness, and training compliance) and five for the procedures and study design (participation willingness, participation rates, loss to follow-up, assessment timescale, and assessment procedures).

These criteria have been adapted for the Tele-UPCAT study and in details measures have been fixed as follows:

Feasibility of Intervention:

- *Acceptability*: intelligibility of rules of the activity in terms of preparation and execution;
- *Training compliance*: duration of the training (at least 3 weeks, that is, the fixed interval of the training);
- *Technical smoothness*: good functioning of the system, defined as the quantity of issues and malfunctioning experienced with the system;
- *Training motivation*: motivation and perceived effort in carrying out the training.

Feasibility of Study Design and Procedures:

- *Participation willingness*: Acceptance of the participation in the study;
- *Participation rates*: Completion of the training (no dropout);
- *Loss to follow-up*: Possibility to collect all data from all outcome measures;
- *Assessment time scale*: Required time for collecting all outcome measures (at least 1 week);
- *Assessment procedures*: Loss to follow-up rates.

The Questionnaire:

To assess the usability and acceptability of the whole therapy, a 32-query questionnaire was created. The questionnaire was tailored for the Tele-UPCAT program, but it is conformed to the standard definitions of usability (22–24) and acceptability (25, 26), respecting items generally evaluated in this kind of assessments.

The questions are divided into four sections composed of eight queries that analyze crucial items of the therapy, from exercises' features to the acceptability of the Actigraphs and enjoyment of the whole therapy. The groups are as follows:

- “Customization of the exercises”: how the participants perceived the exercises as personalized following their abilities and needs, how difficult they were perceived, their preference about the kind of exercise.
- “Acceptability of the Tele-UPCAT system at home in daily life”: how participants coped with a technology system directly installed in their home and the commitment to the therapy in everyday life.
- “Required effort”: whether the participants perceived the whole treatment as tiring and strenuous and the use of Actigraphs every day as bothersome.
- “Suitability of the manual/software”: whether the manual was complete and clear in delivering the instruction to use the system and if the software (including the program) was enjoyable and easy to use, without technical issues.

The questions were structured to be easy to understand for even the youngest participant. When necessary, the parent's perception was taken into account. The questions are followed by a five-point Likert scale, ranging from 1 to 5, where 1 identifies the most negative response and 5 the most positive one. Younger children were helped with a smiley meter to facilitate them in expressing their feelings: they had five illustrated faces with five different expressions, from the saddest to the happiest, and they were asked to indicate the one that better described their thoughts.

Every section of the questionnaire can range from a minimum of 8 (strongly negative) to a maximum of 40 (strongly positive), for a total of 160 for the whole questionnaire, indicating the greatest level of acceptability and usability.

Participants were asked to supply personal thought on every item of the questionnaire to have a wider view on the patient's feedback.

Finally, the wearing time of Actigraphs extracted both from the data and the diary has been considered to have an additional index of acceptability.

### Data Collection

The questionnaire was delivered immediately after the end of the AOT therapy period (T1 or T1 plus) in a face-to-face interview with the patient, with or without the help of the parents. The face-to-face interview allowed the interviewer to explain the queries when necessary and the participant to give their perception more easily.

Daily diaries were also collected to have the data about the wearing time of Actigraphs and parameters about the training time for the analysis of the nine criteria of feasibility.

### Statistical Analysis

Clinical and quantitative data were analyzed by means of the Statistical Package for Social Sciences (SPSS, version 20.0). Median and 95% confidence intervals were calculated. For the questionnaire, raw total score and total for each questionnaire groups and relative percentages were calculated. As a first step, a descriptive analysis of the whole enrolled sample was carried out. Normality of data distribution was verified by Shapiro–Wilk's test, and in relation to the non-normal distribution, we treated the data with nonparametric analyses. In order to assess potential differences between the experiences in the two different age-appropriated software packages (Ubi vs. slides), the Mann–Whitney U independent sample test was performed for the raw scores.

## Results

From the developers' point of view (clinicians and engineers), the Tele-UPCAT platform was designed, realized, and used for its features, judged as optimal at the end of the study: (i) it is possible to present videos and activities based on the developmental needs of each subject; (ii) it can provide a cycle of 15 sessions of daily training, more intensive than the traditional rehabilitative training (which occupies no more than once a week) with low daily duration (no more than 1 h a day), without requiring excessive efforts to be added to the everyday demands of school and other activities; and iii) it is easily transportable and adaptable for in-home use.

### Participants

Participants of this feasibility study were those who were accepted to participate in the wider research project and answered to the *ad hoc* questionnaire, created and developed for the purpose: 29 children and adolescents carried out the Tele-UPCAT training, and all of them filled in the questionnaires on which the present feasibility study is based.

Demographic characteristics of the 29 participants are shown in Table 1. The mean age was 11.73 ± 3.65 years, with a similar number of males and females, 15 and 14, respectively. The affected limb was on the left side in 10 cases and on the right side in 19 cases. According to House Function Classification System (HFCF), five children were in the range between 2 and 3 (meaning a possible hold of the object passively with the affected hand); 13 in the range between 4 and 5 (which means that it is possible to actively stabilize the object with the affected hand); and 11 between 6 and 8 (that is a good or active use of the affected hand).

**Table 1:** Sample characteristics.

Age (year)	Mean	11.73
	SD	3.65
Sex	F	14
	M	15
Affected side	Right	19
	Left	10
HFCS	2–3	5
	4–5	13
	6–8	11

*HFCS, House Function Classification System; SD, Standard Deviation*.

### Training Outcome

All participants completed the training. In details, the mean value of days for achieving the 15 sessions was 20.48 days, ranging from 17 to a maximum of 24, and this was due to the fact that seven patients required more than 21 days (the 3 weeks originally planned) to finish the training.

The wearing time of Actigraph was high in majority of the cases, with a mean of 74.56% and a range from 26.81 to 99.83%.

### Feasibility Outcome

#### Feasibility of Intervention

All four criteria regarding the feasibility of the training intervention were met:

1) All participants figured out instructions both from the printed manual and the software without requiring further explanations and correctly understood goals and ways to proceed each training activity.
2) Overall, all participants reached the criterion of completing at least 80% of the training in 3 weeks; only seven participants completed the whole training in a period ranging from 22 to 24 days.
3) Only two participants experienced a technical issue in the software. These issues were fixed with a phone call with the technical support. One participant had an issue with the camera and it was substituted with a new one. Nevertheless, all participants managed to continue with the training.
4) Regarding the training compliance, all participants showed a positive score (only in one case, just a bit more than neutral) in the “required effort” section of the questionnaire.

#### Feasibility of Study Design and Procedures

All criteria regarding the feasibility of the study design and procedures were met:

5) All eligible participants (100%) agreed to join the project. In three cases, families asked to organize the training in periods in which the required time by the school was reduced (i.e., winter or summer holidays).
6) The totality of enrolled participants agreed to perform the training intervention (i.e., the AOT at home). In one case, a participant started with the SC period (see study design for further details) but did not give the consensus for carrying out the home AOT.
7) All outcome measures (both feasibility and efficacy measures) were collected during assessments and there were no missing data.
8) Follow-up data were collected within a week after the training period, as planned.
9) There was no follow-up loss for any participant who finished the training program; 100% of the participants completed all assessments.

### The Questionnaire

All the 29 children and adolescents gladly accepted the interview or the online questionnaire, adding also some personal comments.

In general, all participants showed a good level of acceptability and usability, with total scores all above 103 points (64.38%). Regarding the four sections: “customization of the exercises” is the one with the lowest range of raw scores (range 18–38); then “acceptability of the Tele-UPCAT system at home in daily life” with a range of 22–38; “required effort” presented a range from 26 to 40; and “suitability of the manual/software" presented a range of 34–40.

There were no differences in the total scores of answers related to the sex of the subjects (*p* > 0.05) and the HFCS level (*p* > 0.05) or the different versions of the software used (Ubi or slides) (*p* > 0.05).

The general opinion of the interviewed sample was globally positive, and there were no significant differences within groups.

Median and 95% confidence interval of scores in the questionnaire (both total and section scores) are shown in Table 2.

**Table 2:** Results of Tele-UPCAT questionnaire.

	**All Sample (*****n*** **=** **29)**		**Ubi (*****n*** **=** **14)**		**Slides (*****n*** **=** **15)**		**Ubi vs. Slides**
	**Median [95% CI]**		**Median [95% CI]**		**Median [95% CI]**		z score^*^ (p)^§^
	**Raw scores**	**%**	**Raw scores**	**%**	**Raw scores**	**%**	
Customization of exercises	32.00 [29.23–33.38]	80.00 [71.23–82.03]	33.00 [31.18–35.15]	80.00 [73.04–86.55]	32.00 [25.31–33.01]	80.00 [63.83–82.54]	−1.642 (0.104)
Acceptability at home	31.00 [29.43–33.00]	77.50 [73.07–81.71]	32.00 [29.72–34.78]	77.50 [73.27–85.47]	31.00 [27.30–32.88]	77.50 [68.24–82.21]	−0.957 (0.347)
Required effort	32.00 [30.96–33.99]	80.00 [76.73–83.49]	33.00 [31.21–35.79]	80.00 [76.77–86.57]	30.00 [29.23–33.49]	75.00 [73.08–83.73]	−1.360 (0.190)
Suitability of manual/software	38.00 [36.78–38.35]	95.00 [91.37–95.58]	38.50 [37.39–31.11]	96.25 [91.88–97.70]	36.00 [35.48–38.16]	90.00 [88.70–95.39]	−1.761 (0.091)
TOTAL	132.00 [127.70–137.43]	82.15 [79.81–85.90]	135.50 [130.56–143.77]	84.69 [81.60–89.86]	130.00 [120.61–134.48]	81.25 [75.38–84.05]	−1.666 (0.104)

* *Mann–Whitney sample test*.

§ *2-sides significant level at 0.05*.

*CI: 95% confidence interval*.

**Figure 2:**
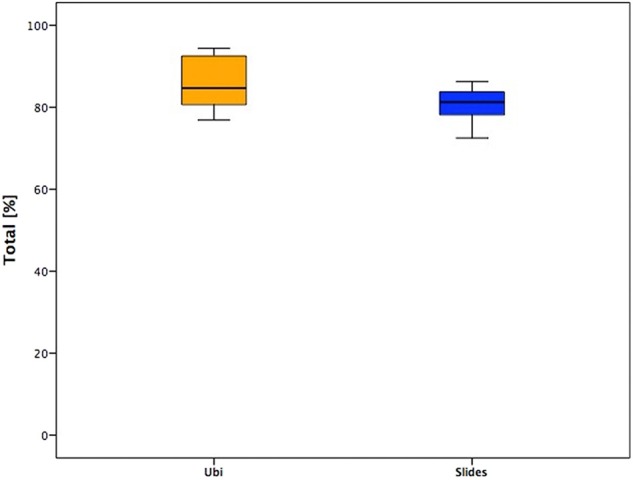
Total answers of the two groups.

Even if the median values between the two age groups were similar, the younger subjects (Ubi) showed larger and higher ranges (Figure 2).

#### Customization of the Exercises

**Figure 3:**
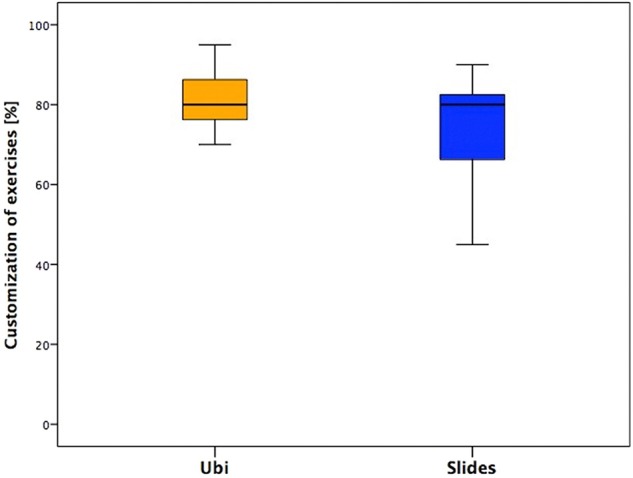
Answers of the section “Customization of the exercises” of the two groups.

This section showed the lowest scores. The median values between the two groups were similar, but the distribution of the answers was toward higher values in younger subjects and lower in older ones (Figure 3).

#### Acceptability of the Tele-UPCAT System at Home in Daily Life

**Figure 4:**
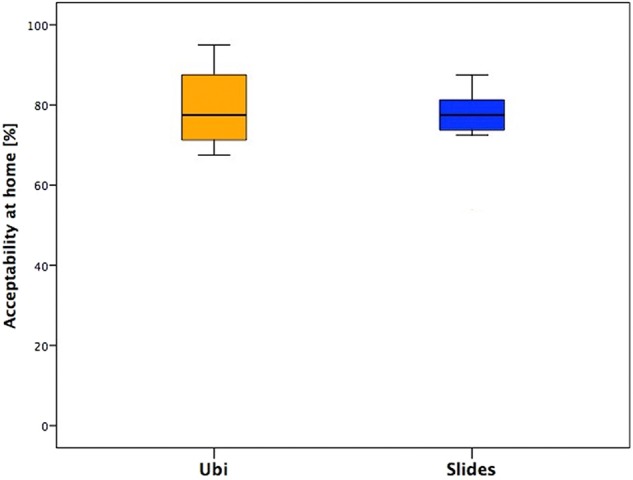
Answers of the section “Acceptability of the Tele-UPCAT system at home in daily life” of the two groups.

In this section, the younger children showed more variability in their answers than adolescents, who gave more similar answers (Figure 4).

#### Required Effort

**Figure 5:**
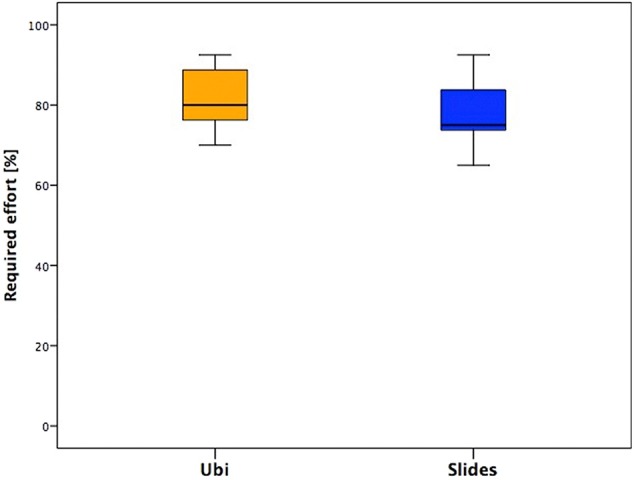
Answers of the section “Required effort” of the two groups.

This section presented similar median values between the two groups, but the distribution is different: in fact, for children aged <12 years (Ubi group), the required effort is perceived as feasible and the tendency of the scores is toward higher values, while for adolescents, the distribution is equal around the median value and globally lower than children's scores (Figure 5).

#### Suitability of the Manual/Software

**Figure 6:**
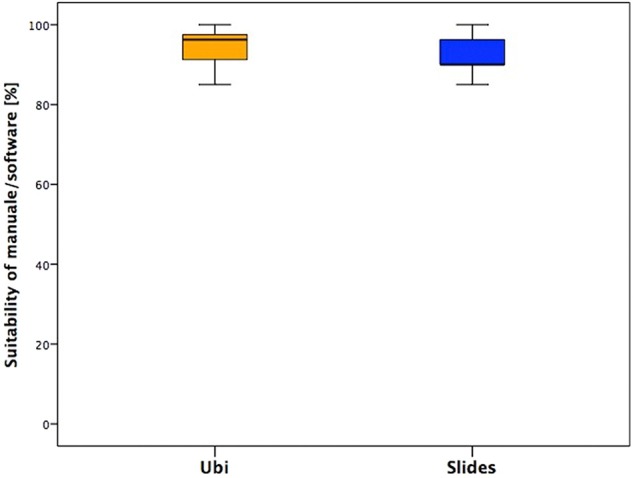
Answers of the section “Suitability of the Manual/Software” of the two groups.

In the last section, one positive aspect is that almost no one needed technical assistance or encountered technical issues during the training, and this supports the stability of the system and its consequent appropriateness for home trainings (Figure 6).

## Discussion

It is widely known that the success of a trial also depends on end users' opinions and satisfaction, which motivate patients to carry out the training and the research staff to improve and optimize the intervention; as a consequence, these two aspects can be positively influenced.

In the present work, we tested the feasibility of a rehabilitative intervention based on the AOT delivered at home, in a sample of children and adolescents with UCP, by the use of an ICT platform. Moreover, we tested also the feasibility of the study design and procedures. To test both these feasibility aspects, in addition to the *ad hoc* questionnaire and data about the wearing time of Actigraphs, a series of previously set and validated measures (21) based on relevant literature (19, 20) have been used.

Based on the data of the number of days to complete the training, the absence of dropouts, and the results of the questionnaires, the Tele-UPCAT platform was demonstrated to be acceptable and usable for home-based training. In addition to this, the platform has shown its stability; in fact, no errors of hardware or software issues have been experienced. In the *ad hoc* questionnaire on training acceptability, most participants indicated a positive commitment to the training program and, together with their parents, they reported high levels of perceived usefulness of the program. For this reason, also the training program can be considered sustainable and relevant, although if we can hypothesize that, weekly contacts with the staff could have a role in sustaining motivation and adherence to the training. This hypothesis has been already reported in the literature (27, 28); in fact, it has been demonstrated how the presence of a tutor could be motivating for participants of a trial. Another explanation could be that in telerehabilitation programs, subjects perceive the planning of competent drivers (i.e., the rehabilitation staff) who remotely guide the training to be behind the training activities. programs, while performed without the technologies, could leave the subjects alone. They can perceive the stress of executing the exercises, while the use of technology can give them the perception that they are executed as in the real rehabilitation setting.

As we included a sample of patients with a wide range of age, the current data can be considered quite representative of the population of children and adolescents with UCP; this suggests that the AOT exercises may be successfully proposed for a telemonitored intervention directly at families' houses. The use of telerehabilitation might increase the accessibility of rehabilitation to a large number of UCP children (e.g., children that live far from the clinical center). It could become cheaper than traditional treatment because telemonitored rehabilitation games could allow the possibility of reducing the “number of children per therapist,” guaranteeing an individualized and intensive training for each patient.

Concerning the feasibility of study design and procedures, we registered a high involvement of participants and families, since all the eligible families accepted to participate. This suggests that families of UCP children and adolescents are highly motivated to introduce ICT platforms for UL rehabilitation at their home.

The participation rate was extremely high, as none of the participants dropped out of the study neither during the AOT home training period nor at follow-up assessments, and also the adherence to the training was very good: only some participants required more than 3 weeks (21 days) to finish the 15 sessions. From their comments, it emerged that this was due to the period of the training execution, i.e., during the school, because during the week, they had many scheduled appointments (therapies, homework, sport, etc.), and they sometimes had a lack of free time to dedicate to the AOT. On the other hand, many participants asked to carry out the training during holidays (Christmas, Easter, and summer) in order to facilitate the organization.

According to the results of the questionnaires, the general opinion of the interviewed sample was globally positive, and there were no significant differences between children (<12 years, Ubi sample) and adolescents (>12 years, Slides sample). This is the first interesting data, because it means that the effort of creating a customized training, which takes care of sex and age preferences, was well-rewarded: all the opinions were coherent and showed appreciation and positive feedback.

When observing the total score of answers, the median values were similar in the two groups, but the score distribution showed higher values in younger subjects. This could be explained because children lived the training in a playful way and performed the exercises with a more motivational software package (Ubi had to achieve missions). This result could also be due to the presence of their parents during the treatment. On the other hand, some adolescents demonstrated to like less the training, and a deeper analysis of the answers of the four sessions gave a clearer view on this aspect.

### Customization of the Exercises

This section showed that the lowest scores with similar values between groups, slightly higher in younger subjects and lower in adolescents.

This could be explained by the fact that the objects selected for the exercises were more suitable for younger children in terms of sizes and features. In fact, the pool of objects and toys has been originally selected to be suitable for a wide range of ages, thinking about making them more compliant for younger children. However, this could mean that sometimes the toys resulted to be not suitable or excessively small for adolescents; moreover, children aged <12 years probably preferred to play with toys, while adolescents could have appreciated something more appropriate for their age and interests. This could clarify the different opinions between the two groups.

In addition, the exercises maybe resulted poorly engaging and boring for adolescents because of their repetitiveness. Despite this, some interesting opinions came from the adolescents. Although they seem to have less appreciated the training, they were more conscious about its relevance, as reported by an example of their personal comments as follows: “I have used some movements of the training in daily life,” “I have used the hand to do certain movements I didn't know I was able to do,” “Now I think about the right movement I've seen during the training and I can do it better,” and “I'm more aware of my hand now.”

### Acceptability of the Tele-UPCAT System at Home in Daily Life

Focusing on the specific answers, more variable in younger children than in adolescents, we noticed that an intensive training at home, which means dedicated time and space for subjects and their family, is feasible but requires organization of home spaces and daily activities. In the children's group, answers' variability is probably related to the weekly routine: there were subjects who had many therapies and other family activities during the days and consequently attending the daily training for them was more demanding; on the other hand, some others were less busy, and the steady commitment of the training represented an appreciated routine.

### Required Effort

This section presented similar median values between the two groups, with a little difference between younger children and adolescents, in fact the second group presented values slightly lower and less variable.

The questions of this section were more focused on the use of Actigraphs and the level of difficulty of exercises. From the free comments in the questionnaire, it emerged that the wristbands were not quite comfortable; some adolescents reported itchiness or being bothered and, in some cases, also embarrassment while wearing Actigraphs in social contexts (school, parties, free time).

Furthermore, the difficulty in performing some actions emerged more in adolescents. It has been explained because they were more aware of exercise movement features, and they reported pain or complaint due to the frequency of the requested movements (done with two intervals of 3 min each).

Several subjects found the duration of the videos excessive, and thus the evaluation of the exercises as boring. They understood the meaning of the two observational intervals, and they demonstrated good levels of attention to the videos, but all of them judged the situation as boring and the videos a little bit too long.

### Suitability of the Manual/Software

In the last section, emerged the stability of the system and its consequent appropriateness for home trainings.

The manual and software were overall considered as clear and complete. Especially the children found the game of their specific software amusing and fun. The slight difference in the answers is basically related to the already reported different software features of the two groups.

## Conclusions

Action Observation Therapy is a new innovative tool that, according to the literature, seems to bring a significant improvement in activity and body function in ICF domains in children and adults. Since the first AOT treatment was carried out by Ertelt et al. (29), the number of studies on adults and children increased and, in this framework, also a new type of innovative therapy, such as the AOT with an ICT platform, has recently been proposed directly at patients' home (30).

We can gladly conclude that, thanks to the presented findings, the home-based AOT is feasible for children and adolescents with UCP. Of course, the next perspective will be to analyze the results of the RCT study in order to understand if this kind of training could have some effects in promoting UL use and performance, immediately after the training and both at the medium and long terms. These parameters will be extracted both from clinical scales and technological measures (Actigraphs).

More and more emphasis should be placed on home-based care and therapies for a number of reasons. Besides cutting costs, this would not only increase efficiency and alleviate the workload of the hospital staff, but also it would offer a wider population the opportunity to avail this treatment. If the ICT solution used in the Tele- UPCAT study was made available, cost-efficient rehabilitation programs could be developed. At the moment, children and young people in non-urban areas are usually at a disadvantage, as often they cannot access treatment easily due to the downsizing or closure of hospitals in their area. The use of technology for rehabilitation also allows an intervention provided remotely and in a non-medical setting, but in a more acceptable and comfortable environment, which is the child's home (14, 31).

Moreover, this type of approach would more than likely reduce family stress, as already demonstrated in another study where a rehabilitative training has been carried out at home with parents (32).

In addition to this, thanks to the remote role of the clinical staff, it is possible, on one hand, to follow in parallel many patients and, on the other hand, to have quantitative results that enrich the clinical data.

In order to investigate also therapists' point of view, it could be useful to develop also a questionnaire addressed to the clinical staff, and this tool is in fact under construction. The analysis of the different end user's perspectives is crucial for ensuring the optimal designing of study protocols and medical devices. There is a growing literature on the development of methods for assessing usability and acceptability of technologies for home-based rehabilitation (33).

From our data, what emerged is that the only disadvantage of an intensive home-based training could be represented by the daily and weekly family routine, as some participants reported to prefer holiday periods to carry out the training. A future perspective could be to organize the 15 sessions in free-time periods (e.g., summer time), perhaps structuring the training without weekend interruption in order to make it last only 2 weeks. On the contrary, another solution could be to shorten the daily training or to provide multiple shorter sections per day in order to guarantee the continuity of the treatment during longer periods. The use of technology gives an advantage of offering multiple and customized solutions in relation to the different rehabilitation needs.

In conclusion, this study demonstrated the feasibility of a home-based AOT with the Tele-UPCAT system in children and adolescents with UCP. The home environment represents an accessible opportunity for rehabilitation among population. Thanks to end users' opinion, the Tele-UPCAT platform can be improved and optimized to further increase its acceptability and usability and, as a consequence, motivation and adherence to the training.

This can, on one hand stimulate, the creation of new platforms for home rehabilitation and, on the other hand, sensitize to follow this methodology for the assessment of the feasibility of the systems to be used for rehabilitation.

The availability of ICT solutions and the rapid progresses of the technology could help to integrate in the Tele-UPCAT system also new hardware and software for better recording of the kinematic aspects of the movement.

Finally, near-future perspective also offers this kind of treatment to other participants, in particular those with bilateral forms of CP, as well as for LL rehabilitation.

## Data Availability Statement

The datasets generated for this study are available on request to the corresponding author.

## Ethics Statement

The studies involving human participants were reviewed and approved by Tuscany Pediatric Ethics Committee (169/2016). Written informed consent to participate in this study was provided by the participants' legal guardian/next of kin. Written informed consent was obtained from the individual(s) for the publication of any potentially identifiable images or data included in this article.

## Author Contributions

GS conceived the idea for this original research, and all other authors contributed to the conception and the design of the study. All authors participated in the design of the study. GS, GC, and AF carried out the enrollment of all children for the study. EB, VM, and GS designed and realized the questionnaire. EB performed all the assessments. VM assisted the families and children within the training. GS performed the statistical analysis. EB prepared the manuscript. GS, VM, GC, and AF read, critically revised, and approved the final manuscript.

### Conflict of Interest

The authors declare that the research was conducted in the absence of any commercial or financial relationships that could be construed as a potential conflict of interest.
